# Infection with COVID-19 promotes the progression of pancreatic cancer through the PI3K-AKT signaling pathway

**DOI:** 10.1007/s12672-023-00842-9

**Published:** 2023-12-08

**Authors:** Xusheng Zhang, Bendong Chen, Kejun Liu, Yongxin Ma, Yimin Liu, Hongcai Zhou, Peng Wei

**Affiliations:** 1 Ningxia Medical University, Yinchuan, 750004 China; 2 General Hospital of Ningxia Medical University, Yinchuan, 750004 China

**Keywords:** COVID-19, Pancreatic cancer, Machine learning, PI3K-AKT signaling pathway, Support vector machine, Random forest tree

## Abstract

**Objective:**

To investigate the effect of COVID-19 infection on pancreatic cancer.

**Methods:**

Based on the mRNA-Seq data of COVID-19 patients and pancreatic cancer (PC) patients in the GEO database, we used a support vector machine (SVM), LASSO-Cox regression analysis and random forest tree (RF) to screen the common signature genes of the two diseases and further investigate their effects and functional characteristics on PC, respectively. The above procedures were performed in R software.

**Results:**

The proteins COL10A1/FAP/FN1 were found to be common signature genes for COVID-19 and PC, were significantly up-regulated in both diseases and showed good diagnostic efficacy for PC. The risk model based on COL10A1/FAP/FN1 showed good PC risk prediction ability and clinical application potential. Tumor typing based on COL10A1/FAP/FN1 expression levels effectively classified PC into different subtypes and showed significant differences between the two subtypes in terms of survival prognosis, immune levels, immune checkpoint expression levels, mutation status of common tumor mutation sites, and drug sensitivity analysis. While pathway analysis also revealed that FN1 as an extracellular matrix component may be involved in the biological process of PC by regulating the PI3K-AKT signaling axis.

**Conclusion:**

The upregulated expression of COL10A1/FAP/FN1, the characteristic genes of COVID-19, are potential diagnostic targets for PC, and the upregulated expression of FN1 may promote the progression of PC by activating the PI3K-AKT signaling pathway. The COL10A1/FAP/FN1-based typing provides a new typing approach for PC, and also provides a good reference and idea for the refinement of PC treatment and subsequent clinical research.

## Introduction

Since the first case of coronavirus disease (COVID-19) was diagnosed, the disease has spread widely worldwide and the ongoing pandemic has led to a wide range of social, economic, and health problems, among which health issues are of particular concern to the general public. Successive studies have found that the COVID-19 epidemic has produced a series of direct and indirect health problems in different populations, such as psychological problems and physical disorders [1–3]. It also has an important impact on some patients with pre-existing underlying diseases, leading to the progression and modification of the patients’ underlying diseases.

Pancreatic cancer (PC) is one of the most malignant tumors in the digestive system and has one of the worst prognoses, with a 5-year survival rate of about 10%. The morbidity and mortality rates of PC are increasing year by year in China [4, 5]. The difficulty of treating PC is exacerbated by its high heterogeneity, aggressiveness, metastasis, and drug resistance [6]. Up to now, the etiology and mechanisms of PC have not been fully elucidated. However, with continuous in-depth research, it has been found that various factors such as obesity and genetics are related to the occurrence and development of the disease. Studies at the molecular level have demonstrated that the pathogenesis of pancreatic cancer is accompanied by a large number of genetic mutations, which [7–9].

During the COVID-19 epidemic, there were a series of effects on patients with underlying diseases that led to rapid disease progression. However, there appears to be limited research on the effects of COVID-19 infection on cancer, and we also did not obtain systematic statistical results on the promotion of pancreatic cancer development by COVID-19 infection. Nevertheless, increased mortality in patients with pancreatitis during COVID-19 infection has been reported [10], and pancreatic transplant patients are more likely to develop diabetes, resulting in a poorer prognosis [11]. Based on these findings, this study aimed to explore the association between COVID-19 infection and pancreatic cancer by analyzing the blood gene sequencing results of COVID-19 patients combined with RNA-Seq data from pancreatic cancer patients.

## Materials and methods

### Data collection and processing

This study obtained gene expression profiling data related to COVID-19 and pancreatic cancer from the public database GEO (https://www.ncbi.nlm.nih.gov/geo/), including COVID-19 datasets (GSE171110 and GSE152418, there are 54 samples and 34 samples, respectively) and pancreatic cancer datasets (GSE15471 and GSE62165, there are 78 samples and 131 samples, respectively). These data are all from public databases and have no usage restrictions, we followed the data access policy and publication guidelines of the GEO database. The statistical analysis of the measured data between groups was performed using the Wilcoxon rank-sum test with R software.

### Feature gene selection

Using the “limma” package in R software, we analyzed the differentially expressed genes in COVID-19 and pancreatic cancer and drew heat maps and volcano plots. The up-regulated proteins were preliminarily screened using SVM-RFE in the “e1071/kernlab” package. The interaction genes were further evaluated and ranked for their importance using the “randomForest” package to select feature genes. A P < 0.05 is considered statistically significant.

### Model construction and subtype analysis

In R software, we used the “pROC” package to analyze the diagnostic efficacy of COVID-19 feature genes in pancreatic cancer and drew diagnostic ROC curves. Risk models were constructed and validated using the “rms” and “rmda” packages, and subtype analysis was performed using the “ConsensusClusterPlus” package. Survival analysis of subgroups was performed using Cox regression analysis in the “survival” package [12].

### Immune correlation analysis

Using Spearman correlation analysis, we performed simple immune correlation analysis on measurement data that did not conform to normal distribution. The correlation coefficient was calculated to evaluate the degree of association between genes related to immunity in the gene expression profile data.

### Signal pathway analysis

**Fig. 1:**
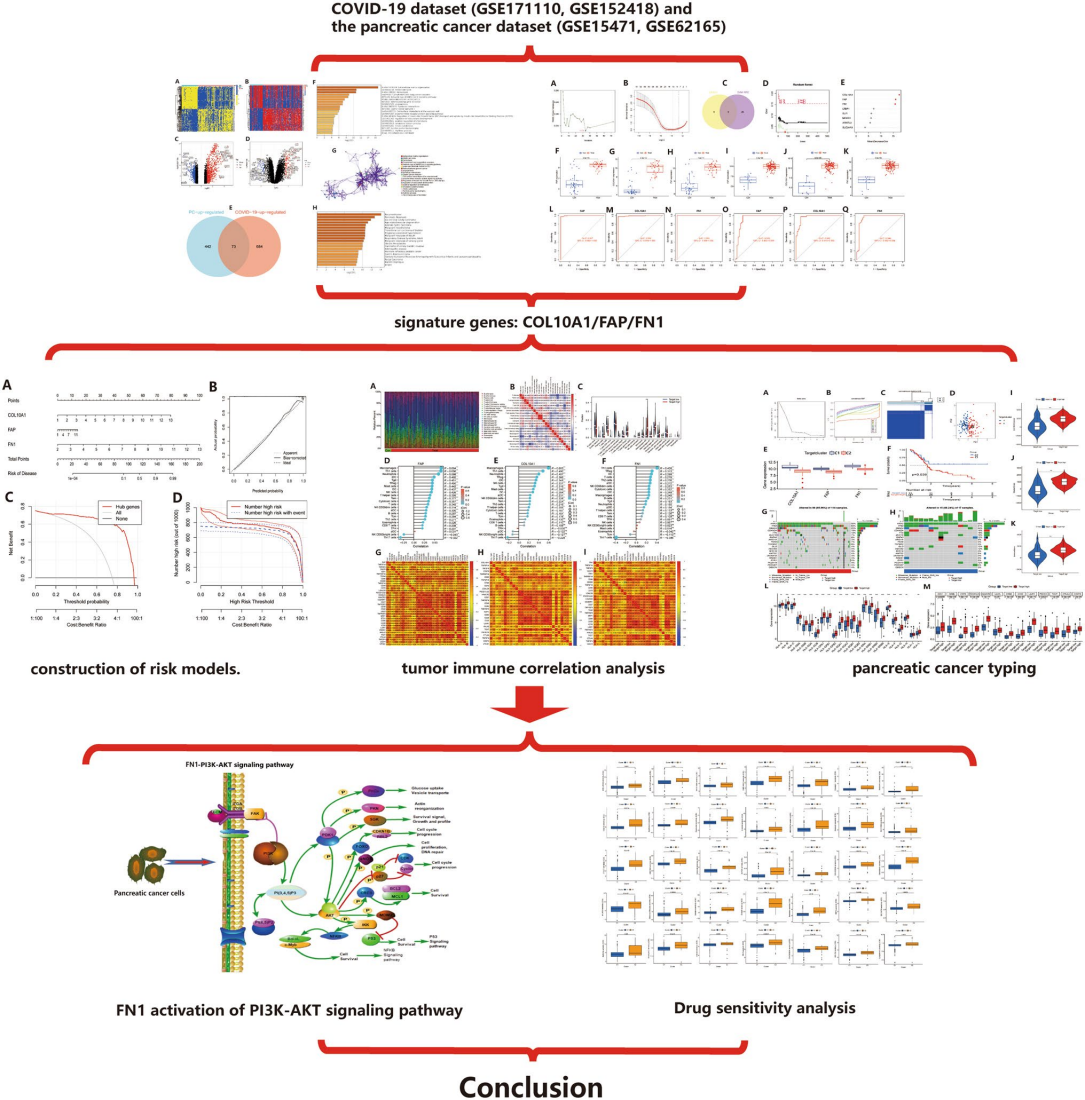
The summary of images from this study

To investigate whether COVID-19 infection promotes the progression of pancreatic cancer through the PI3K-AKT signal pathway. Based on the feature genes in the gene expression profiling data, we further explored their enrichment in specific signal pathways and determined the functional and regulatory networks of these signal pathways in pancreatic cancer. The overall research approach of the study is demonstrated in the Fig. 1.

## Results

### Differentially expressed gene screening and functional/pathway analysis

**Fig. 2:**
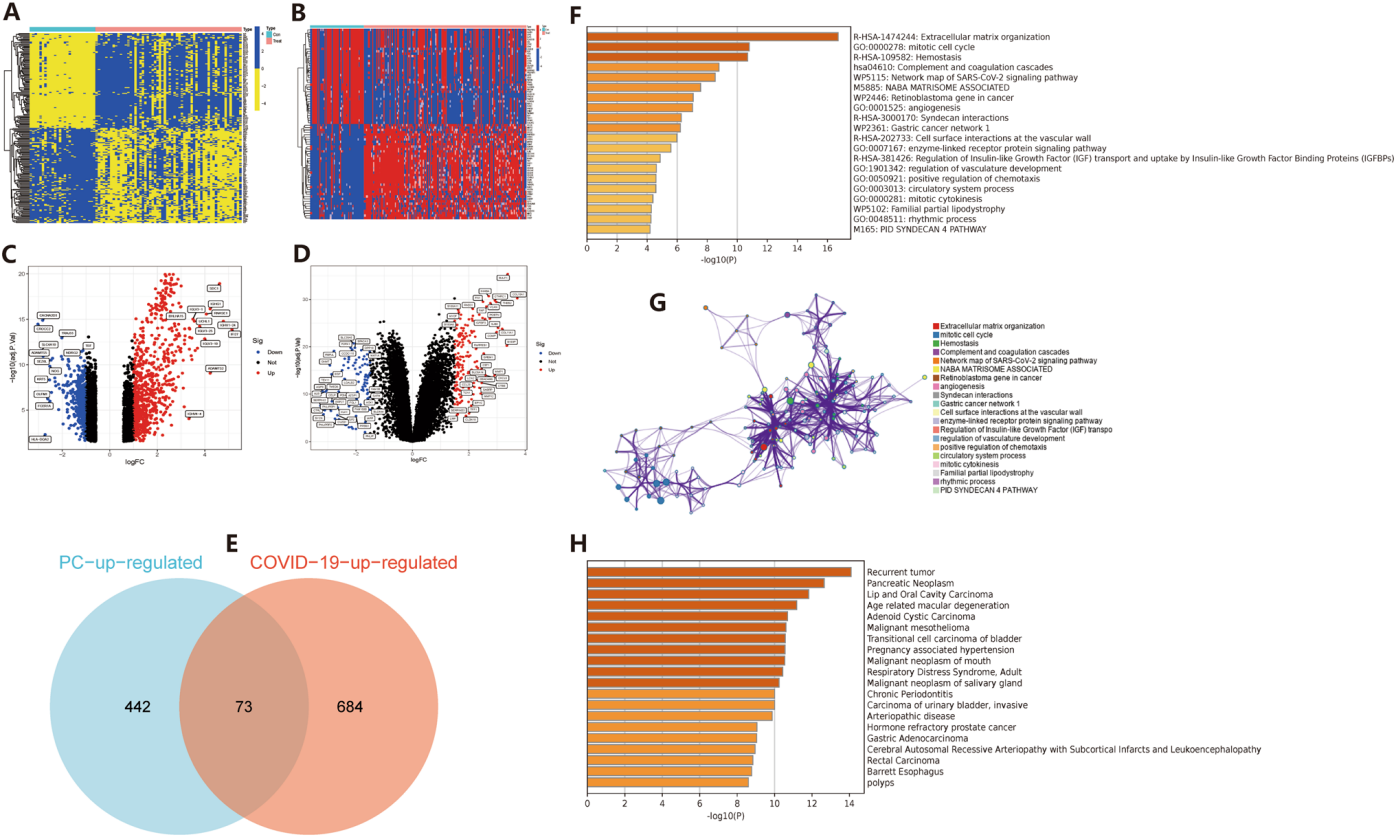
Differentially expressed gene screening and functional/pathway analysis. **A**, **B** Heat map of differentially expressed gene screening for two diseases, sequentially COVID-19, PC. **C**, **D** Volcano map of differentially expressed genes, sequentially COVID-19, PC. **E** Wayne plot of the intersection of up-regulated proteins expressed in both diseases. **F**, **G** Functional and pathway analysis, **F** is a bar graph and **G** is a network relationship graph. **H** Bar graph of disease-specific enrichment analysis

Based on the mRNA-Seq data of 88 COVID-19 patients in the GEO database (GSE171110, GSE152418), and 209 pancreatic cancer patients in GSE15471 and GSE62165, we screened differentially expressed genes separately. We showed that 1201 genes were significantly differentially expressed in COVID-19 patients, including 757 upregulated genes (logFC ≥ 1, P < 0.05). In PC, there were 745 genes that were significantly differentially expressed, including 515 upregulated proteins (logFC ≥ 1, P < 0.05). The heat map (Fig. 2A, B) and volcano map (Fig. 2C, D) of differentially expressed genes for the two diseases were plotted separately. Then, we performed an intersection of the upregulated genes in both diseases, and the results showed that 73 proteins were significantly upregulated in both diseases (Fig. 2E). We then analyzed the functions of the 73 significantly upregulated proteins and evaluated the major diseases in which they were enriched. The results indicated that these genes were mainly enriched in functional areas such as mitochondrial cell cycle, angiogenesis, enzyme-linked receptor protein signaling pathway, regulation of vasculature development, positive regulation of chemotaxis, circulatory system process, mitotic cytokinesis, rhythmic process, and others. The WikiPathways were mainly enriched in the network map of the SARS-CoV-2 signaling pathway, retinoblastoma gene in cancer, and other pathways (Fig. 2F, G). Additionally, the disease ontology (DO) enrichment analysis showed that these proteins are primarily involved in recurrent tumors, pancreatic tumors, adenoid cystic carcinoma, malignant mesothelioma, and other diseases (Fig. 2H).

### Screening for genes with common characteristics of COVID-19 and PC

**Fig. 3:**
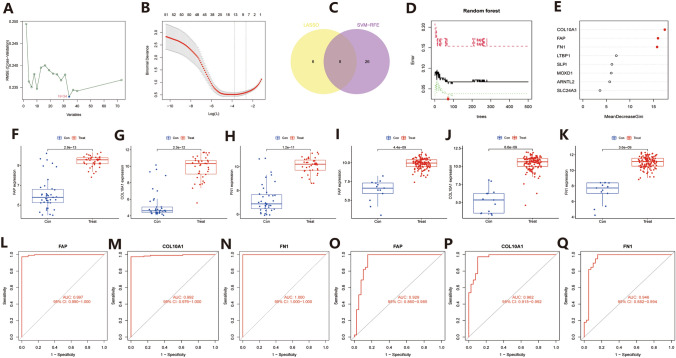
Feature gene screening. **A** Support vector machine(SVM) screening of feature genes. **B** LASSO-Cox regression analysis to screen the feature genes. **C** Venn diagram of the intersection of support vector machine(SVM) and LASSO-Cox regression analysis screening results. **D**, **E** Random forest tree (RF) plots of the intersection results and their corresponding importance ranking bubble plots. **F**–**H** Differential expression box plots of COL10A1/FAP/FN1 in dataset GSE15471. **I**–**K** Differential expression box plots of COL10A1/FAP/FN1 in dataset GSE62165. **L**–**N** Diagnostic ROC curves of COL10A1/FAP/FN1 on PC in dataset GSE15471. **O**–**Q** Diagnostic ROC curves of COL10A1/FAP/FN1 against PC in dataset GSE62165

We then further screened 73 intersecting up-regulated proteins in both diseases. Based on the expression levels of upregulated proteins in PC, we first performed a machine learning screening of the included proteins using a support vector machine (SVM-RFE. Setting parameters: seed = 123; Ganna = 0.1; Cost = 1; Accuracy: 0.708), which showed minimal cross-validation when the result was 34, thus screening out 34 upregulated proteins (Fig. 3A). Next, we further analyzed the 73 included proteins using LASSO-Cox regression analysis(Setting parameters: seed = 123; Nfolds = 10), which showed that 14 proteins were screened out when the cross-validation result was minimal (Fig. 3B). Then we again intersected the results screened by the two different machine learning methods, and the results showed that 8 proteins were selected in both machine learning methods (Fig. 3C). To ensure the credibility of the results, we again analyzed the screened intersection genes using the random forest tree learning method and ranked the importance of the 8 included proteins, then selected the top 3 proteins for subsequent analysis. The results showed that the RF Ntree (Setting parameters: seed = 123; Ntree = 500; RfGenes=[1:3]) took the minimum error at position 72, and the importance bubble plot showed that COL10A1/FAP/FN1 were the top 3 proteins in terms of importance, respectively (Fig. 3D, E). Then, COL10A1/FAP/FN1 were defined as the common signature genes of COVID-19 and PC in this study.

Next, we further determined the expression levels of the three signature genes based on the expression levels of COL10A1/FAP/FN1 in PC, and the results showed that the expression levels of COL10A1/FAP/FN1 were significantly upregulated in dataset GSE15471 (Fig. 3F–H, logFC > 1.5, P < 0.001), and the results were validated using dataset GSE62165, which also showed that the expression of COL10A1/FAP/FN1 was significantly upregulated compared to the paracancerous tissue (Fig. 3I–K, logFC > 1.5, P < 0.001). In this study, the diagnostic efficacy of the three signature genes for PC was also analyzed, and the results showed good diagnostic efficacy of COL10A1/FAP/FN1 for PC (ROC-AUC > 0.9) in both datasets GSE15471 (Fig. 3L–N) and GSE62165 (Fig. 3O–Q).

### Construction and validation of the nomogram risk model

**Fig. 4:**
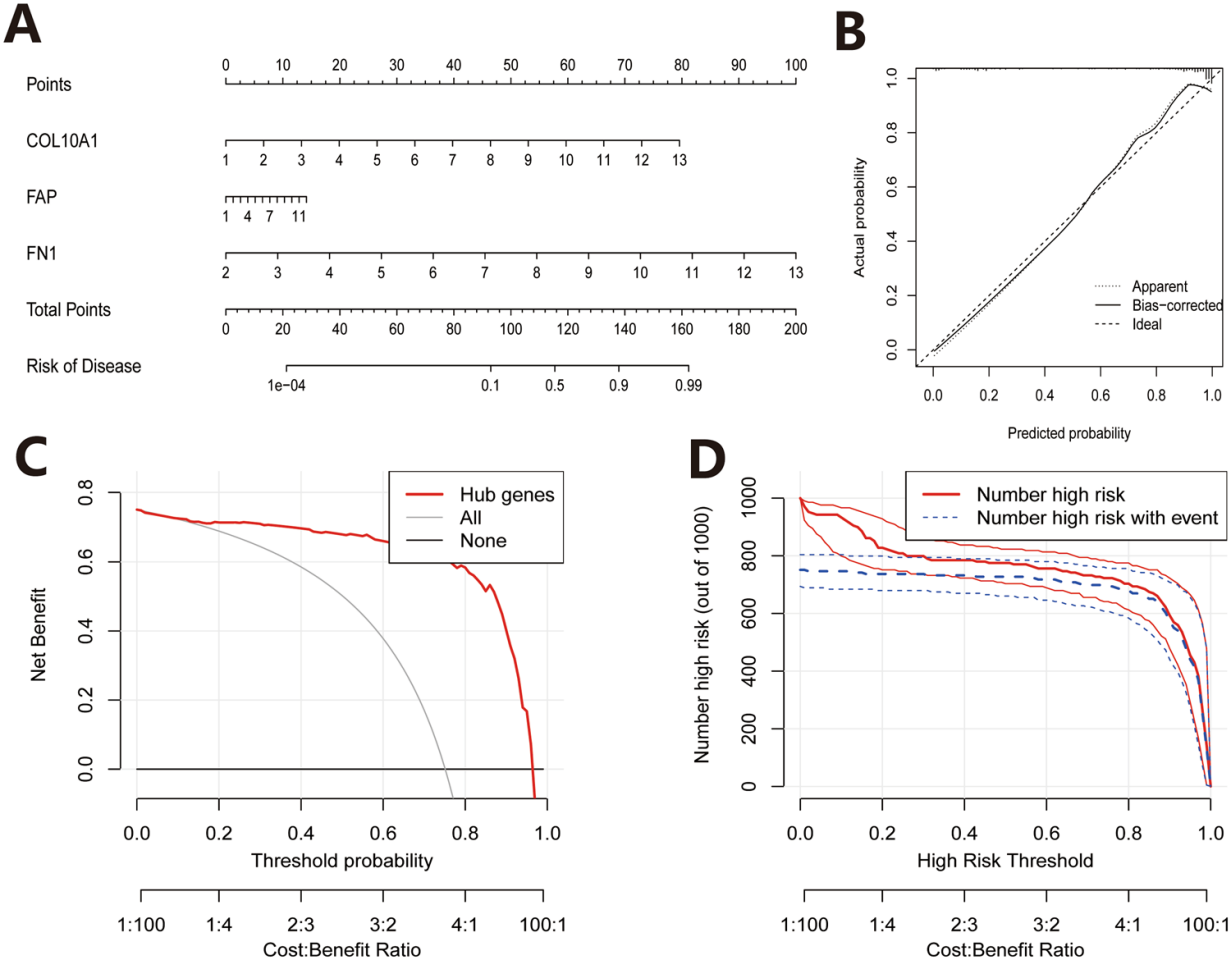
Construction and validation of risk models. **A** The nomogram risk model is based on COL10A1/FAP/FN1 expression. **B** Calibration curve. **C** Clinical decision curve (DCA) of the model. **D** Clinical impact curves of the model

We found that the expression levels of COL10A1/FAP/FN1 were significantly upregulated in PC and showed good diagnostic efficacy for PC, suggesting that these three proteins are diagnostic targets for PC. Based on the expression levels of COL10A1/FAP/FN1, we further constructed a nomogram risk model for pancreatic cancer. From this risk model, we can see that the upregulation of COL10A1/FAP/FN1 expression is a risk factor for pancreatic cancer, and the upregulation of all three increases the risk of developing PC in patients (Fig. 4A). Then, to verify the efficacy of the model, we plotted the calibration curves, which calculates the predicted probability for each observation based on the model, divides them into 10 groups based on increasing predicted probabilities, and then calculates the incidence rate for each group by comparing the predicted outcomes from the model with the actual observed outcomes. The scatter plot is created by plotting the corresponding incidence rates on the y-axis against the predicted probabilities on the x-axis. The results showed that the calibration curves displayed a good fit, suggesting that the risk model has good predictive efficacy (Fig. 4B). The clinical applicability of the risk model was then analyzed, and the decision curve (DCA) showed a good net clinical benefit (Fig. 4C), and the positive detection rate of the model in the clinical impact curve was also very close to the number of real positive patients, further supporting the predictive effectiveness of the model (Fig. 4D). Taken together, the PC risk model based on the characteristic genes COL10A1/FAP/FN1 has good predictive efficacy and clinical application potential.

### Subtyping of pancreatic cancer and its correlation analysis

**Fig. 5:**
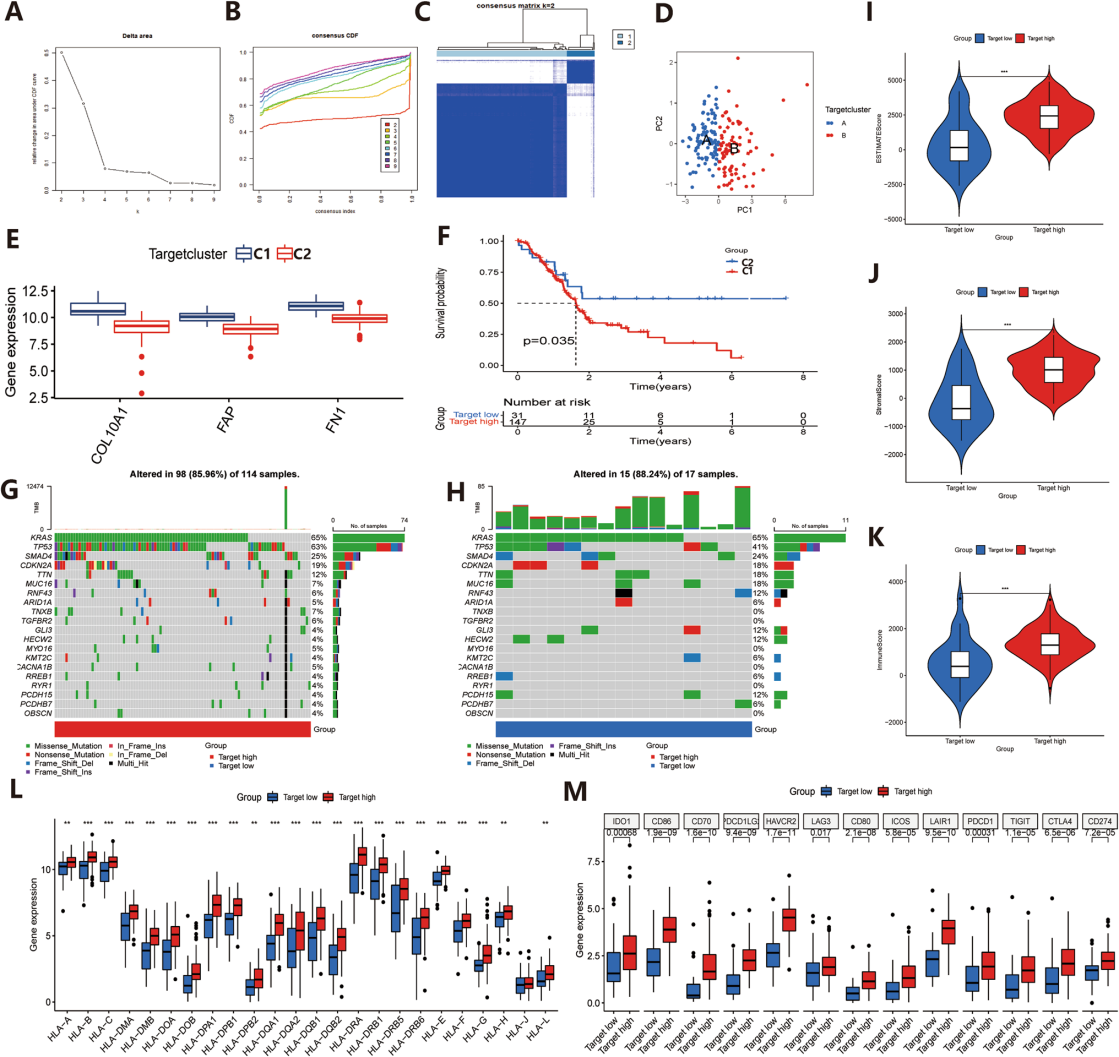
Signature gene-based pancreatic cancer typing and its correlation analysis. **A**–**C** Results of unsupervised cluster analysis in PC based on COL10A1/FAP/FN1 expression levels. **D** Principal component analysis (PCA) scatter plots to validate the clustering analysis results. **E** Box plots of expression analysis of the signature genes in the two subtypes. **F** Prognostic analysis between the two subtypes. **G** Mutation status of common tumor mutation loci in C1 subtypes. **H** Mutation status of common tumor mutation loci in C1 subtypes. **I**–**K** Violin plots of the results of tumor microenvironment analysis between the two subtypes. **L** Box plot of leukocyte factor expression between the two subtypes. **M** A box plot of the expression of common immune checkpoints between the two subtypes

Next, in this study, PC was subtyped using unsupervised cluster analysis based on the expression levels of COL10A1/FAP/FN1. The results showed that the included samples effectively scored into two subtypes, C1 and C2 (Fig. 5A–C). To verify the validity of the typing, we also performed principal component analysis (PCA) on the included samples. The results demonstrated that the samples were clearly divided into two groups, A and B, which was consistent with the cluster analysis results, confirming the validity of the typing (Fig. 5D). Furthermore, we analyzed the expression of the three characteristic genes between the two subtypes. The results revealed that COL10A1/FAP/FN1 were highly expressed in the C1 subtype and less expressed in the C2 subtype. All three genes exhibited the same expression trend between the two subtypes (Fig. 5E). We also investigated the survival differences between the two subtypes. The results showed a significant difference in survival between the C1 and C2 subtypes, P = 0.35. (P < 0.05), with the C1 group associated with a poorer prognosis and the C2 group with a better prognosis (Fig. 5F). In addition, we analyzed the mutation of common tumor mutation genetic loci between the CI and C2 subtypes. The results revealed that the mutation rate between both subtypes was over 85%. Furthermore, several mutation loci such as TP53, TNX8, TGFBR2, MYO16, and CACNA18 displayed significantly higher mutation rates in C1 (signature gene high expression group) compared to C2 (signature gene low expression group). This suggests that C1 has higher mutation rates at certain mutant loci (Fig. 5G, H). We then evaluated the tumor microenvironment of the two subtypes. The results indicated significant differences in the immune score, stromal score, and overall score of the tumor microenvironment between the C1 and C2 subtypes, indicating marked dissimilarities in the tumor immune microenvironment (Fig. 5I–K, P < 0.001). Based on these results, we further analyzed the expression levels of HLA factors between the two subtypes. The results demonstrated remarkable differences in the expression of HLA factors, such as HLA-A, HLA-DMA, HLA-DMB, HLA-DOA, HLA-DOB, HLA-DPA1, HLA-DPB1, HLA-DPB2, HLA-DQA1, HLA-DQA2, HLA-DQB1, HLA-DQB2, HLA-DRA, HLA-DRB1, HLA-DRB5, HLA-DRB6, HLA-H, and HLA-L, between C1 and C2 (Fig. 5L, P < 0.005). Additionally, we analyzed the expression of common immune checkpoints between the two subtypes. The outcomes revealed marked differences in the expression of various immune checkpoints, including classical immune checkpoints such as CD274, PDCD1, and CTLA4 (Fig. 5M, P < 0.001).

### Tumor immune correlation analysis

**Fig. 6:**
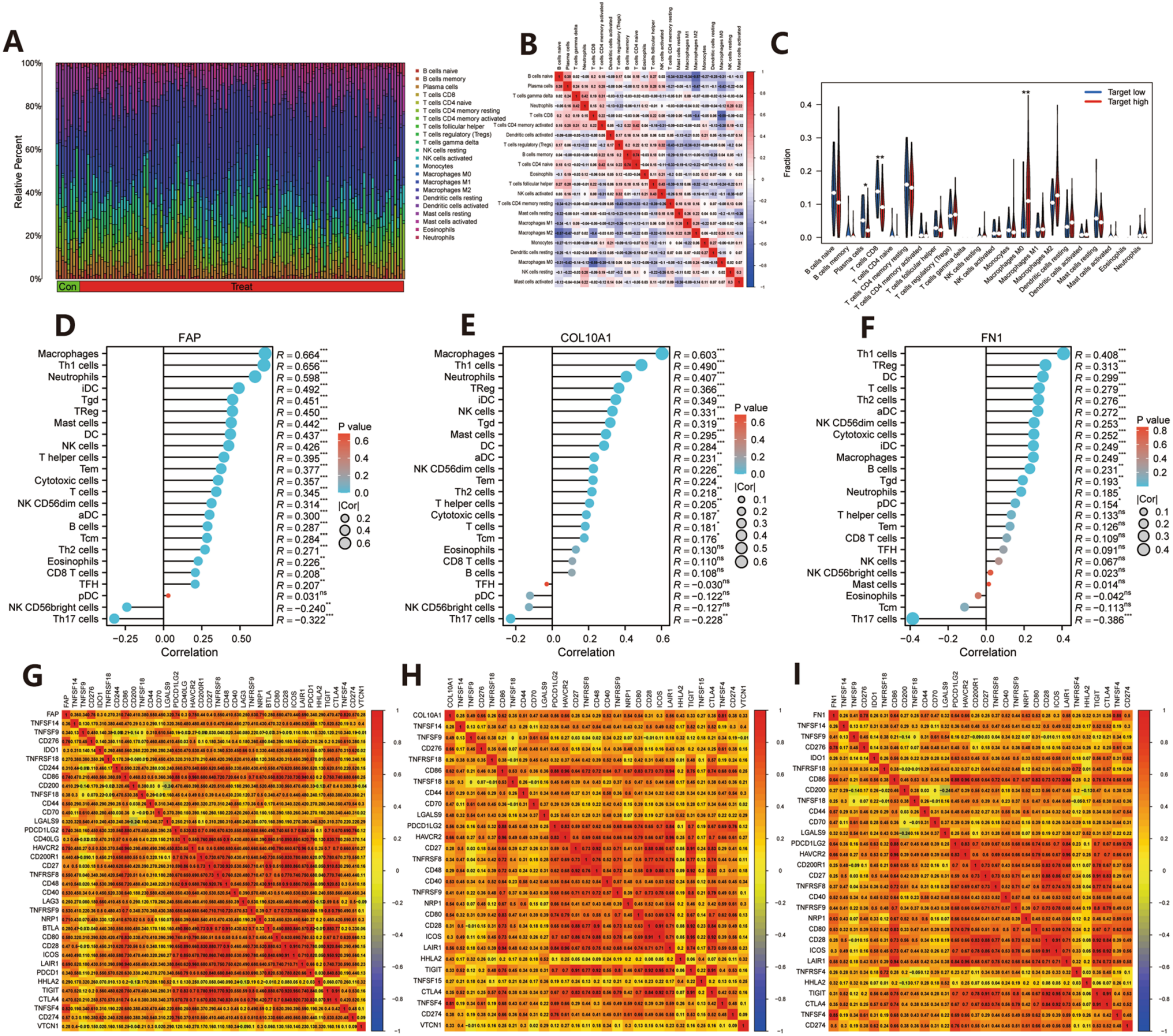
Tumor immune correlation analysis. **A** Bar graph of the composition of tumor immune cells in paracancerous tissue and PC. **B** Heat map of the correlation analysis between each tumor immune cell. **C** Violin plot of the difference analysis between the levels of tumor immune cells in paraneoplastic tissues and PC. **D**–**F** Bar graphs of correlation analysis between FAP/COL10A1/FN1 and each tumor immune cell in order. **G**–**I** Heat map of correlation analysis between FAP/COL10A1/FN1 and each immune checkpoint, in order

Next, the tumor microenvironment of the entire pancreatic cancer (PC) was analyzed in this study. We compared the composition of each immune cell in the tumor microenvironment between paraneoplastic tissues and PC (Fig. 6A), as well as the relationship between them (Fig. 6B). The results revealed that the levels of TFH, resting NK cells, monocytes, macrophages M0, macrophages M1, macrophages M2, resting mast cells, and activated mast cells were remarkably different in paraneoplastic tissues and PC. The expression levels of TFH, resting NK cells, monocytes, and resting mast cells in PC were significantly lower than those in paraneoplastic tissues, while the aggregation levels of macrophages M0, macrophages M1, macrophages M2, and activated mast cells in PC were higher than those in paraneoplastic tissues, and there was a negative correlation among most tumor immune cells (Fig. 6C, P < 0.05). We then analyzed the correlation between COL10A1/FAP/FN1 and tumor immune cells, and the outcomes showed that FAP had significant positive correlations with macrophages, Th1 cells, neutrophils, iDC, Tgd, TReg, mast cells, and NK cells (Fig. 6D, R > 0.4, P < 0.001). COL10A1 showed significant positive correlations with macrophages, Th1 cells, and neutrophils (Fig. 6E, R > 0.4, P < 0.001). FN1 mainly showed a significant positive correlation only with Th1 cells (Fig. 6F, R > 0.4, P < 0.001). In combination with the analysis results, COL10A1/FAP/FN1 were mainly positively correlated with most of the tumor immune cells, which might be associated with an enhanced tumor immune response. Based on the previous significant differences in the expression levels of the two subtypes of C1 and C2 immune checkpoints, we further investigated the correlation between COL10A1/FAP/FN1 and tumor immune checkpoints. The results showed that FAP had significant positive correlations with CD276, CD86, CD200, CD44, CD70, PDCD1LG2, HAVCR2, CD200R1, CD27, TNFRSF8, CD48, CD40, TNFRSF9, NRP1, CD80, CD28, ICOS, LAIR1, TIGIT, CTLA4, TNFSF4, CD274, and other immune checkpoints (Fig. 6G, R > 0.4). COL10A1 also showed marked positive correlations with some immune checkpoints, such as TNFSF9, CD276, CD86, CD44, CD70, LGALS9, PDCD1LG2, HAVCR2, CD40, TNFRSF9, NRP1, CD80, LAIR1, HHLA2, and TNFSF4 (Fig. 6H, R > 0.4). FN1 mainly showed significant positive correlations mainly with TNFSF9, CD276, CD86, CD44, CD70, PDCD1LG2, HAVCR2, CD40, TNFRSF9, NRP1, CD80, LAIR1, TNFSF4, and CD274 (Fig. 6I, R > 0.4).

### Signaling pathway analysis of FN1

**Fig. 7:**
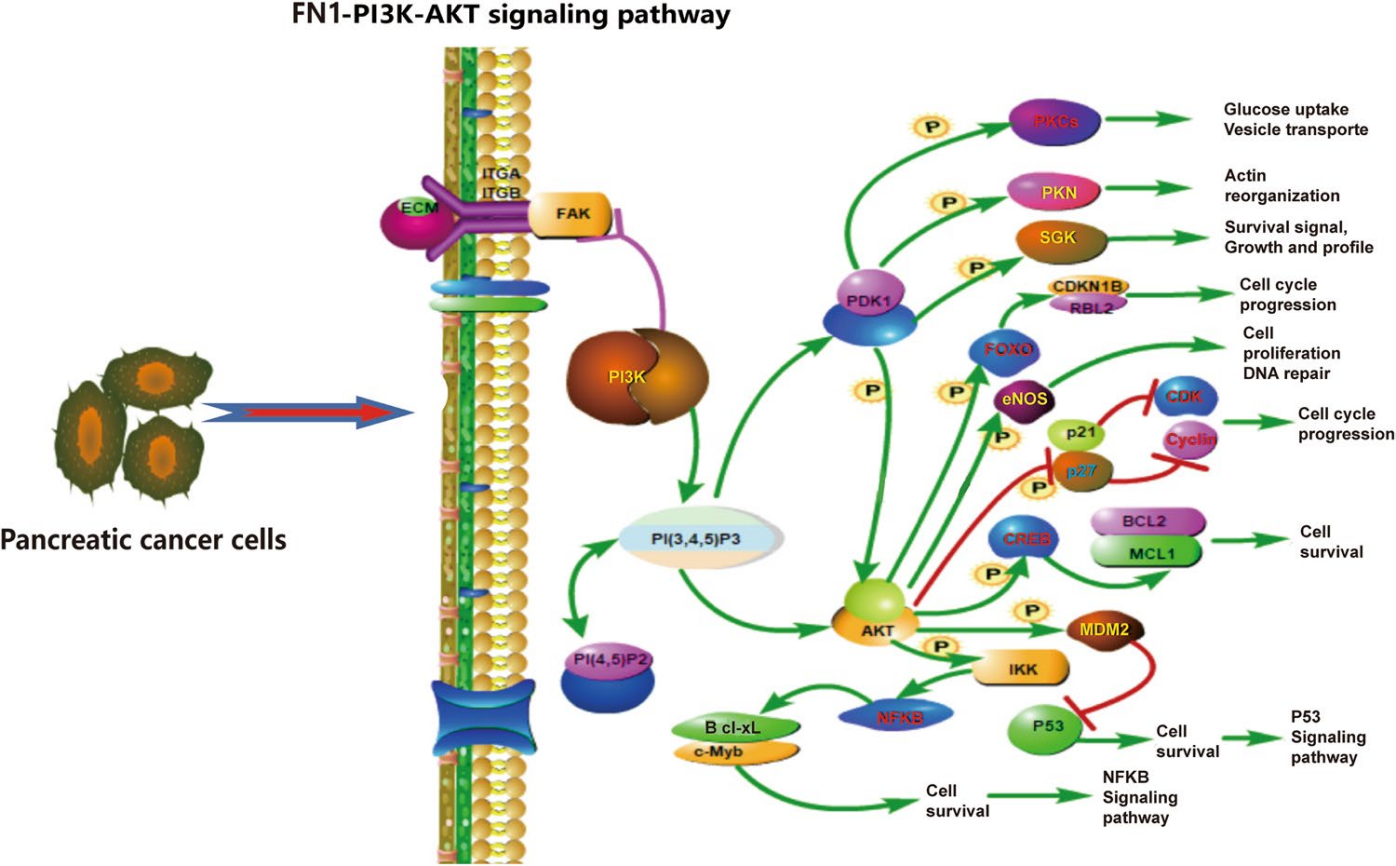
Schematic representation of FN1 activation of PI3K-AKT signaling pathway

This study also investigated the signaling pathways involved in COL10A1/FAP/FN1, and the results showed that FN1 is a component of the extracellular matrix (ECM), which is a characteristic gene for cancer. The ECM components and factors can activate the PI3K-AKT signaling pathway through integrins ITGA and ITGB, thereby affecting the cell cycle and regulating the signaling process in cancer. This process is closely related to the proliferation and apoptosis of cancer cells. Based on this, we hypothesize that the upregulation of FN1 expression in PC may promote the progression of pancreatic cancer and is associated with a poorer prognosis for patients (Fig. 7).

### Drug sensitivity analysis between C1 and C2 subtypes

**Fig. 8:**
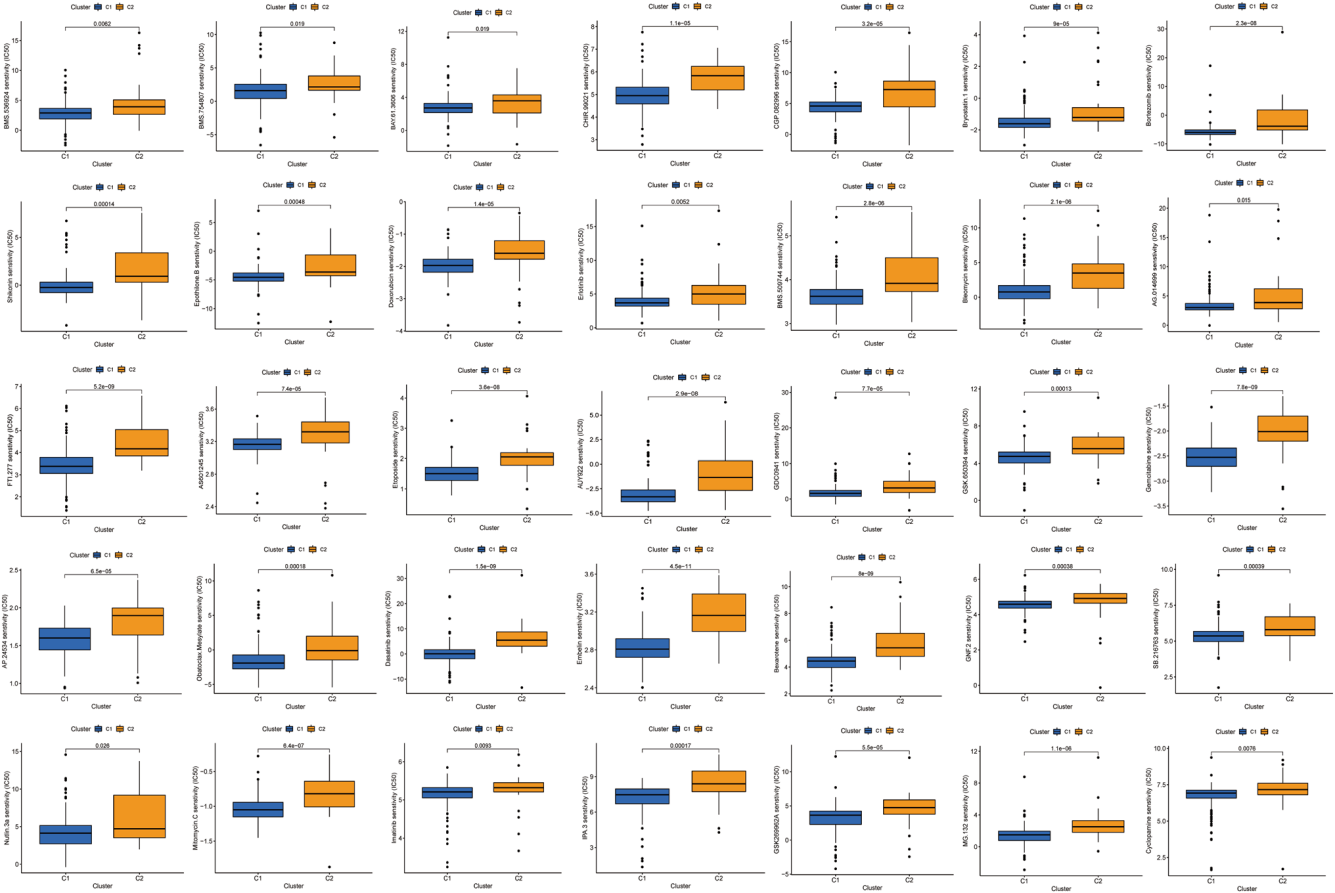
Box plot of the drug sensitivity analysis between C1 and C2 subtypes

At the end of the study, the IC50 value was calculated based on the pRRophetic algorithm and signature genes expression profile to predict the sensitivity of different chemotherapeutic drugs between C1 and C2 subgroups. The results showed that the C1 subgroup exhibited higher sensitivity to most drugs compared to the C2 subtype (Fig. 8). Taken together, the results of the typing method and drug sensitivity analysis presented in this study may provide more targeted drug therapy for PC patients. However, further clinical trials are needed to demonstrate the feasibility of the study’s results.

## Discussion

The widespread dissemination of COVID-19 has had a significant impact on both the physical and mental health of the general public. However, the impact of COVID-19 on cancer patients has been largely ignored due to the diversion of public attention toward more acute and prominent health problems. In this study, we screened for common characteristic genes of COVID-19 and PC based on a machine learning method and explored the effect of COVID-19 infection on PC patients. As a result, we found that three proteins, namely COL10A1/FAP/FN1, may play an important regulatory role in PC during COVID-19 infection.

Type X collagen α 1 chain (COL10A1), a member of the collagen family, is a gene associated with the progression of a variety of human tumors. It has been shown that upregulation of COL10A1 expression is significantly associated with the survival prognosis of breast cancer patients, and knockdown of COL10A1 significantly inhibits proliferation, migration, and invasion of breast cancer cells, as well as promotes apoptosis [13]. In addition, it has been mentioned that in breast cancer, COL10A1 promotes cell proliferation, migration, and invasion by targeting prolyl 4-hydroxylase β polypeptide (P4HB) [14]. In gastric cancer, COL10A1 expression was also found to be significantly increased compared to non-tumor specimens, and it was significantly associated with tumor T stage and pathological stage. Patients with high COL10A1 expression had a worse survival prognosis [15]. It was also found that overexpression of COL10A1 promoted cell migration and invasion in cervical cancer, and silencing of COL10A1 inhibited cell proliferation, metastasis, and epithelial mesenchymal transition in cervical cancer by inactivating TGF-β/Smad signaling [16]. Studies on COL10A1 in pancreatic cancer remain scarce, with only one relatively recent study mentioning that the COL10A1-DDR2 axis promotes the progression of pancreatic cancer by regulating MEK/ERK signaling [17]. In addition, COL10A1 has also been studied in lung adenocarcinoma and colorectal cancer [18, 19].

Fibroblast activation protein alpha (FAP), which is also a cancer signature gene, is involved in extracellular matrix degradation and many cellular processes, including tissue remodeling, fibrosis, wound healing, inflammation, and tumor growth. It has been mentioned that FAP is one of the characteristic genes of gastric cancer and is associated with patient prognosis [20]. Similarly, FAP was found to be expressed at higher levels in advanced ovarian cancer, and silencing FAP induced apoptosis. Further studies found that FAP is critical for ovarian cancer cell survival by sustaining NF-kB activation through recruitment of PRKDC in lipid rafts [21]. Moreover, FAP also has prognostic significance in colorectal cancer, and further analysis showed that FAP in central tumors combined with tertiary lymphoid structures has more prognostic value in predicting recurrence after radical resection [22]. Another study found aberrant overexpression of FAP on cancer-associated fibroblasts, suggesting that FAP and Nectin4 could serve as promising targets for safe and effective CAR-T therapy in malignant solid tumors [23].

Fibronectin 1 (FN1) encodes fibronectin, a glycoprotein that exists as a soluble dimer in the plasma and as a dimer or multimeric form on the cell surface and in the extracellular matrix. The encoded preprotein undergoes protein hydrolysis to generate mature proteins. Fibronectin is involved in cell adhesion and migration processes, including embryogenesis, wound healing, coagulation, host defense, and metastasis. This gene is also considered a cancer signature gene. High expression of FN1 correlates with advanced pathological stage, T-stage, N-stage, and M-stage, predicting a poor prognosis in breast cancer patients. Silencing of FN1 impairs the proliferation and invasive ability of breast cancer cells, suggesting that FN1 may serve as a prognostic marker and therapeutic target for breast cancer [24]. FN1 has also been identified as a prognostic marker and therapeutic target for papillary thyroid microcarcinoma (PTMC) and may have potential as a diagnostic biomarker [25]. In gastric cancer, high expression of FN1 is associated with reduced overall survival (OS) [26]. Furthermore, in pancreatic cancer, high expression of FN1 has been linked to poorer survival prognosis and it may serve as a key signaling gene for therapeutic intervention in pancreatic cancer [27].

The expression levels of COL10A1, FAP, and FN1 in PC maintained the same trend and were all significantly upregulated, indicating good diagnostic efficacy for PC and potential diagnostic targets. Additionally, high expressions of COL10A1, FAP, and FN1 in the nomogram model were identified as risk factors for PC. The risk model based on COL10A1, FAP, and FN1 also demonstrated good predictive efficacy and potential clinical application. During the clinical treatment process we can only test the levels of COL10A1, FAP, and FN1 in patients, and based on these levels, use our constructed risk model to predict the patient’s risk of illness, thus leading to more precise diagnosis and treatment planning. However, further testing and analysis are needed to validate its clinical utility and promote its application.

Then, based on the expression levels of COL10A1, FAP, and FN1, this study conducted a subtype analysis of PC. The included samples were divided into two subtypes, C1 and C2, and the expression levels of COL10A1, FAP, and FN1 in the two subtypes were analyzed. The results showed that C1 corresponded to the higher group of the three proteins, while the expression levels of the three proteins in the C2 group were lower. There was a significant difference in survival prognosis between the two subtypes, with the C1 subtype having a significantly poorer survival prognosis than the C2 subtype. Some mutation sites that were focused on also exhibited higher mutation rates in C1, suggesting that mutations in these sites may be one of the factors influencing the poorer prognosis of C1 subtypes. Further analysis of the immune microenvironment of the two subtypes revealed significant differences in immune scoring, matrix scoring, and overall scoring between the Target high group (C1) and the Target low group (C2). C1 subtypes had significantly higher scores than C2, suggesting that the immune microenvironment of C1 subtypes has a higher immune response and increased matrix components. This study also investigated the expression of leukocyte factors and common immune checkpoints between the two subtypes, and the results showed that C1 subtypes had higher levels than C2 subtypes. This finding suggests that leukocyte function and immune checkpoint levels were both upregulated in C1 subtypes, which could provide a new research direction for the precise treatment of PC by regulating leukocyte factors or targeting highly expressed immune checkpoints. Moreover, given the drawbacks of the current single-targeted drugs, new multi-targeted drugs can be successfully developed based on this study, which can provide a reference for the research of targeted therapy for PC.

Further immune correlation analysis also suggested that the expression levels of TFH, resting NK cells, monocytes, and resting mast cells in PC were significantly lower than those in paracancerous tissues, whereas the aggregation levels of macrophages M0, macrophages M1, macrophages M2, and activated mast cells in PC were higher than those in paracancerous tissues, and the combined effect of various tumor immune cells played a decisive role in the overall tumor immunity. The expression levels of COL10A1/FAP/FN1 were mainly positively correlated with most of the immune cells, FAP is significantly positively correlated with macrophages, Th1 cells, neutrophils, iDCs, Tgd cells, TReg cells, mast cells, and NK cells; COL10A1 is significantly positively correlated with macrophages, Th1 cells, and neutrophils; FN1 is mainly only significantly positively correlated with Th1 cells. The immune response of the tumor is determined by the complex interplay of numerous immune cells, and the heterogeneity of immune cells and the complex immune evasion mechanisms of the tumor make tumor immunity highly complex. Through this study’s simple analysis, We can tentatively speculate that the overexpression of COL10A1/FAP/FN1 is positively correlated with cancer immunity. And the immune response of PC was mainly enhanced with the increase of COL10A1/FAP/FN1 expression levels. And in the immune checkpoint correlation analysis, the three proteins also showed high correlation with different immune checkpoints, respectively, and the expression levels of COL10A1/FAP/FN1 could be used to predict the expression levels of immune targets and thus the sensitivity of targeted therapies for pre-treatment reference when the treatment involving some relevant immune targets is involved.

Next, we analyzed the main pathways involved in COL10A1, FAP, and FN1 and found that FN1, as one of the extracellular matrix (ECM) components, could activate the PI3K-AKT signaling pathway through the integrins ITGA and ITGB. This activation can affect the cell cycle and regulate the signaling process in cancer, which is one of the important pathways in the cancer signaling pathway [28–30]. Based on this, it is hypothesized that COVID-19 infection further promotes the upregulation of FN1 expression, which in turn activates the PI3K-AKT signaling pathway and promotes the progression of PC. This hypothesis may be associated with poorer survival prognosis in patients.

Then, based on the typing results of COL10A1, FAP, and FN1 expression levels, we also analyzed the sensitivity of common chemotherapeutic and targeted drugs between the two subtypes. The results showed that various drugs generally had higher sensitivity in the C1 subtype, suggesting that these drugs are more promising for the C1 subtype. However, further clinical studies are needed to prove the objectivity and feasibility of the results of this analysis.

This study found that COL10A1/FAP/FN1 are significantly differentially expressed genes in PC and have good diagnostic and predictive capabilities. In COVID-19 infected patients, COL10A1/FAP/FN1 are also significantly upregulated, and FN1 may promote the progression of pancreatic cancer through the PI3K-AKT signaling pathway. Although the overall trend of the COVID-19 pandemic has significantly declined, we still encounter a considerable number of patients infected with COVID-19, including those with PC in clinical practice. With the advancement of sequencing technologies, genetic sequencing has become increasingly feasible. Firstly, by examining the expression levels of the COL10A1/FAP/FN1, we can sensitively detect whether COVID-19 infection is promoting cancer progression. Secondly, using a nomogram model, we can predict the risk of disease progression in pancreatic cancer. Furthermore, by conducting subtype grouping of patients, we can tailor treatment strategies to be more targeted. Lastly, based on the analysis of drug sensitivity, we can further develop new drugs with higher sensitivity, providing additional therapeutic options for PC. The correlation between COL10A1/FAP/FN1 and different immune checkpoints was analyzed, which can be used to predict the levels of related targets based on their expression levels, providing reference for the research of new targeted therapies for PC.

Lastly, we need to declare that this study has limitations. The analyzed data is derived from different datasets, resulting in potential biases due to variations in sequencing time and methods. Secondly, this study tended to select upregulated genes and did not include downregulated genes for analysis. This may result in neglecting biological functions associated with downregulated genes. Additionally, the conclusions of this study are based on the analysis of public database data, which may be subject to confounding factors. Therefore, further evidence from evidence-based medicine is needed to support these findings.

## Conclusion

The upregulated expression of COVID-19 signature genes COL10A1/FAP/FN1 are potential diagnostic targets for PC, and FN1 may affect apoptosis and other processes through activation of PI3K-AKT signaling pathway to promote PC progression. The clustering typing based on the expression levels of COL10A1/FAP/FN1 of COVID-19 signature genes provides a new typing approach for PC, enabling a more refined analysis of PC and providing a good reference for the sophisticated treatment of PC and further clinical studies.

## Data Availability

The data used in this study were obtained from the GEO (https://www.ncbi.nlm.nih.gov/geo/) database, both of which are available in publicly available databases. This study complies with its data use and publication rules.
